# Conference Report: 1997 WIRELESS COMMUNICATIONS CONFERENCE Boulder, CO August 11–13, 1997

**DOI:** 10.6028/jres.102.047

**Published:** 1997

**Authors:** Roger B. Marks, Michael S. Heutmaker

**Affiliations:** Electromagnetic Fields Division, Electronics and Electrical Engineering Laboratory, National Institute of Standards and Technology, 325 Broadway, Boulder, CO 80303-3328; Lucent Technologies, P.O. Box 900, RM2-2063, Princeton, NJ08542-0900

## 1. Introduction

Wireless telecommunications systems, exploiting the air as a transmission medium, provide portability and are increasingly taking the place of wired systems even for fixed terminals. However, the air is a shared medium. Compatibility and standards problems are central to the technological issues faced by this industry.

In many countries, notably the United States, wireless communications licenses are being awarded without strict reference to their use and without interoperability standards. The result is a somewhat chaotic state in which many transmission standards coexist. Without government to dictate standards, systems operators must make independent decisions, based largely on technological grounds, concerning their equipment standards. The wisdom of such decisions depends, to large extent, on the quality and detail of technical data available about proposed systems.

The desire to explore some of the finer details of wireless systems was behind the first Wireless Communications Conference, held in Boulder, Colorado in 1996. The great response to this conference led directly to the 1997 Wireless Communications Conference (WCC’97), also held in Boulder, on August 11–13, 1997. WCC’97 was sponsored by the Microwave Theory and Techniques Society of the Institute of Electrical and Electronics Engineers (IEEE) and by the International Microelectronics and Packaging Society (IMAPS). Technical cosponsors of the event were the National Institute of Standards and Technology (NIST), the Institute for Telecommunications Sciences (ITS), and the IEEE Communications Society. There were 254 people registered for the conference, an increase of about 50 % from 1996. Attendees came from 14 nations: Austria, Belgium, Canada, Finland, France, Germany, Israel, Japan, Korea, the Netherlands, Spain, Switzerland, the United Kingdom, and the United States; 42 registrants were from outside the United States. Both the 1996 and 1997 conferences were chaired by Dr. Roger Marks of NIST.

One of the essential features of the conference is that is offers a deep view of the industry, from high-level issues such as systems to low-level topics such as components, in a single-track format. As a result, it brings together customer and supplier throughout the supply chain in an interactive format. Attendees have voiced their approval of this opportunity to see the big picture and help address the many wireless communications problems that require an interdisciplinary approach.

### 2. Technical Sessions

The Technical Program was overseen by the Technical Chair, Dr. Michael S. Heutmaker of Lucent Technologies, along with an international Technical Program Committee of 23 members. From 81 submitted manuscripts from 17 countries, this committee selected 51 for oral presentations. These were arranged in 10 sessions:

- *Local Multipoint Distribution System (LMDS)* was chaired by Mohammad Shakouri of Hewlett-Packard. LMDS is a wireless system providing broadband digital access for video, voice, and data to homes and business. The system has received a great deal of attention abroad. Furthermore, with the United States Federal Communications Commission (FCC) having recently allocated over 1 GHz of bandwidth between 27.5 GHz and 31.3 GHz for such services, many of the major telecommunications players are rushing to establish themselves in this potentially huge marketplace. The session included papers covering system issues, propagation, and active and passive components. Doug Gray of Hewlett-Packard Wireless Systems (Cupertino, CA) presented two talks. An invited talk described the company’s proposed utilization of the broadband local access spectrum at 28 GHz. The second talk illustrated the impact of path loss, hub spacing, etc., on subscriber coverage and infrastructure cost in the planned deployment of an LMDS network. Propagation in residential areas at 28 GHz is an important issue for LMDS systems, and was the subject of two talks. Peter Papazian of the Institute for Telecommunication Sciences (Boulder, CO) described how trees and other scatterers cause time-varying path loss and depolarization at 28 GHz. In addition to path loss and coverage data, Aly Elrefaie of Hewlett-Packard (Cupertino, CA) presented initial results from a planning tool that uses aerial photographs to evaluate coverage vs. antenna height and subscriber position within the cell. Zaher Bardai of Hughes Aircraft Co. (Torrance, CA) described monolithic microwave integrated circuit (MMIC) process technology tradeoffs to make millimeter-wave devices more affordable. Peter Petre of Hughes Research Labs (Malibu, CA) showed measurement and simulation results on mm-wave passive devices.
- *Wireless Systems and Modems* was chaired by Kari-Pekka Estola of Nokia Research Center and included papers on Si chip sets, pager system performance, and an adaptive-rate cellular modem. Tsuneo Tsukahara of NTT System Electronics Laboratories (Kanagawa, Japan) described the performance of a log/limiting amplifier, quadrature modulator, and demodulator (Si bipolar) and a dielectric root-Nyquist filter, intended for use in giagahertz-band intermediate frequency (IF) applications. Millimeter-wave systems such as LMDS tend to use IFs in the gigahertz range. Jay Jacobsmeyer of Pericle Communications Co. (Colorado Springs, CO) presented simulations of a modem that can vary its data rate according to the wireless channel conditions, and can achieve an average data rate exceeding 28.8 kbit/s during a single time division multiple access (TDMA) time slot. This scheme would enable a cellular modem to achieve data rates comparable to wireline connections, instead of the 9.6 kbit/s rate presently supported in North American TDMA. Tom Sexton of Motorola (Fort Worth, TX) showed that the beating between simulcast transmissions in a high data rate paging system can limit coverage unless the system is optimized to minimize delay spread throughout the coverage area. Finally, Jeff Durec of Motorola (Tempe, AZ) described a chip set including Si bipolar transmitter, receiver preamplifier, and receiver integrated circuits (ICs), and a BiCMOS baseband IC for 900 MHz cordless phone applications.
- *Systems and Signal Processing*, chaired by Modest Oprysko of IBM, contained an invited paper on wideband code division multiple access (CDMA) and contributed papers on a proposed digital demodulator architecture, signal processing methods, and the performance of narrow-beam antennas in a cellular system. Fumiyuki Adachi of NTT DoCoMo (Kanagawa, Japan) gave an invited talk describing system architecture and field tests of the NTT wideband CDMA system. The paper focused on a fast cell search algorithm that enables asynchronous cell site operation, and other features that enable flexibility for the system to support various wideband data services. Mitsuru Uesugi of Matsushita (Yokohama, Japan) presented simulations to show that a demodulator architecture that combines an adaptive equalizer and forward error correction can outperform conventional architectures by several decibels under some conditions. Andreas Schmidbauer of the Technical University of Munich described simulations and experiments to validate a new algorithm for equalizing the multipath of a wireless channel, without the need for the communication protocol to include training bits for the equalizer. Hiroyuki Tsuji of the Communications Research Laboratory (Yokohama, Japan) discussed simulations to show that a new scheme using cyclostationarity to find the direction of arrival (DOA) of signals outperforms conventional algorithms. Mark Reudink of Metawave Communications Corp. (Redmond, WA) showed results from a field test in a rural area which found an 8 dB to 10 dB improvement of signal to noise- and distortion (SINAD) ratio when a narrow-beam smart antenna system was compared to an omnidirectional antenna configuration.
- *Characterizing Digital Modulation* was chaired by Seng-Woon Chen of QUALCOMM and included papers on the error vector, amplifier distortion, and CDMA simulation. Ben Zarlingo of Hewlett-Packard (Everett, WA) showed that an adaptive equalizer can compensate for the effects of linear distortion in a measurement of the error vector of a digitally modulated signal. Michael Heutmaker of Lucent Technologies (Princeton, NJ) showed how nonlinear distortion from a power amplifier is manifested in error vector measurements. Joseph Staudinger of Motorola (Tempe, AZ) presented simulations of a power amplifier to investigate how gain compression and phase distortion each contribute to the intermodulation distortion and spectral regrowth at the amplifier output. Roland Hassun of Hewlett-Packard (Santa Rosa, CA) described error vector methods to characterize transmitter performance. Reza Mahmoudi of the Delft University of Technology (Delft, The Netherlands) presented some simulation results on the performance of CDMA systems.
- *Device Nonlinearity*, chaired by Masami Akaike of the Science University of Tokyo, featured papers spanning the range from system-level performance to semiconductor device fabrication issues. John Sevic of QUALCOMM (San Diego, CA) described how the talk time of a CDMA mobile is largely independent of the power amplifier efficiency (measured at maximum output power). Because of the power control in the CDMA system, a mobile actually transmits at full power only rarely, which makes the efficiency at maximum power irrelevant to the average battery drain. Hsin-Chin Chang of QUALCOMM (San Diego, CA) presented theoretical and experimental results on a method to predict the adjacent-channel power ratio of a CDMA amplifier from the two-tone intermodulation distortion of the amplifier. Joseph Staudinger of Motorola (Tempe, AZ) described simulations to explore issues in the modeling of spectral regrowth of amplifiers from measurements of gain compression and phase distortion. David Kinzel of ATN Microwave (N. Billerica, MA) showed that a predictive model for the dependence of the output power and efficiency on termination impedance (in a transistor amplifier) can be developed using data from an automated load-pull system. Gene Tkachenko of Alpha Industries (Woburn, MA) presented experimental results on GaAs MESFETs (metal-Schottky field effect transistors) fabricated in various geometries, which show that the intermodulation performance varies with the gate recess width, which is the distance between the side of the gate electrode and the nearby wall of the recess in the semiconductor.
- *Active Devices* was chaired by Joseph Staudinger of Motorola and contained papers on device modeling, a low-supply-voltage, low noise amplifier design, and a GaAs MMIC transceiver. Lutfi Albasha of the University of Leeds (Leeds, UK) described a quasi-two dimensional device model that combines breakdown mechanisms and thermal effects for the purpose of simulating millimeter-wave power amplifiers. Matthias Rittweger of the Institut für Mobil- und Satellitenfunktechnik (IMST, Kamp-Lintfort, Germany) showed how the packaged device large-signal modeling problem may be broken into two manageable parts—data from a chip-on-board characterization is used to develop large-signal device models, and the package is simulated in three dimensions to develop an equivalent circuit. Urs Lott of the Swiss Federal Institute of Technology (Zurich, Switzerland) described a GaAs MESFET low noise amplifier designed for a supply voltage of 0.9 V. The amplifier achieves 10 dB gain and 2.7 dB noise figure at 2 GHz, and draws 2 mA supply current. Tina Quach of Motorola (Tempe, AZ) described a GaAs transceiver MMIC for wireless local area network (LAN) applications at 3 V which contains a diversity switch, transmit/receive switch, low noise amplifier, downconversion mixer, and power amplifier. The chip is packaged to fit in the PCMCIA (Personal Computer Memory Card International Association) format.
- *Ceramic Packaging and Devices*, chaired by Charles Hodges of Flextronics, contained papers on design and fabrication issues in ceramic packaging and on electrically-tunable filters. Hisayoshi Wada of Kyocera (Kagoshima, Japan) described a ceramic package design method using a combination of 3D simulation and S-parameter simulation. Test circuits were measured and simulated up to 40 GHz. Peter Barnwell of Heraeus Cermalloy (Conshohocken, PA) described features of two commercial thick-film technologies: low-temperature co-fired ceramic (LTCC) and subtractive thick film. Olli Salmela of the Nokia Research Center (Helsinki, Finland) presented simulation results (up to 40 GHz) concerning an unexpected increase in the coupling between two striplines when a row of vias is placed between the striplines. John Estes of Motorola (Albuquerque, NM) showed an example of the use of LTCC technology for embedded passive components in a receiver design. The ceramic circuit contained a T/R switch, four filters, matching network, bias circuitry, and a low-noise amplifier. Young-Soo You of the Sunmoon University (Asan City, Korea) described how a combination of dielectric resonators and microactuators enables the center frequency or the bandwidth of a radio frequency (RF) filter to be tuned over a frequency range of up to 20 %.
- *Integrated Passive Components*, chaired by Peter Petre of Hughes Research Labs, included papers on passive component design in thin-film and thick-film technologies, micromachined filters, and an MMIC ring coupler design. Donald Benson of Flextronics (San Jose, CA) presented results on the use of thin-film circuits to integrate resistors, inductors and capacitors for designs up to 5 GHz. Simulations and measurements agreed within about 0.1 dB for a spiral inductor and two bandpass filters. Vijai Tripathi of Oregon State University (Corvallis, OR) illustrated design procedures for multilayer passive circuits such as spiral inductors and coupled line filters. Scott Raby of the University of Arizona (Tucson,AZ) described simulations to analyze the effect of a perforated ground plane (used in some LTCC processes) on characteristic impedance, phase velocities, coupler design, and filter design. Andrew Brown of the University of Michigan (Ann Arbor, MI) showed how silicon wafer processing is used to fabricate a filter bank at 10 GHz. The integration of varactor diodes in the design enables such filters to be electrically tunable. Hongming An of Nortel (Ottawa, Canada) presented an MMIC hybrid coupler design at 38 GHz, intended for use in mixer designs for wireless LAN or LMDS.
- *Wireless LAN and Indoor Propagation*, chaired by Roger Dalke of the Institute for Telecommunication Sciences (ITS), contained papers on a proposed systems architecture as well as on measurement and modeling of indoor propagation. Tsutomu Takeuchi of Kyoto Sangyo University (Kyoto, Japan) presented simulation results to show that the combination of a return-to-zero modulation format (PSK-RZ) and artificial frequency selective fading might enable bit rates of 5 Mbit/s to 10 Mbit/s for indoor applications. Gamantyo Hendrantoro of Carleton University (Ottawa, Canada) described measurements of multipath for indoor propagation at 29.5 GHz, including the effects of changing the angle of the receiving antenna. Christopher Holloway of the Institute for Telecommunication Sciences (Boulder, CO) showed that a simple model for the decay rate of the impulse response of the propagation within a room depends on the room geometry and reflection coefficient of the walls. This simple model reproduces the trends observed in measured data and sophisticated models. Hakan Inanoglu of Omnipoint Corp. (Colorado Springs, CO) described how a combination of the ray-tracing method and the parabolic equation method is useful in the simulation of indoor propagation characteristics.
- *Antennas and Propagation*, chaired by Christopher Holloway of the Institute for Telecommunication Sciences (ITS), contained papers on antenna design, man-made noise, simulations, and the performance of array antennas in cellular systems. James McLean of the University of Wisconsin (Madison, WI) described two antenna designs: a double-tuned monopole and dielectric-loaded, partially folded bowtie. Mark Yaklich of the University of Wisconsin (Madison, WI) explained some design considerations for an antenna system consisting of a monopole and four notch antennas. Roger Dalke of the Institute for Telecommunication Sciences (Boulder, CO) showed that recent measurements of man-made noise at 137 MHz differ significantly from predictions based on standard methods. Robert Achatz, also of ITS, presented some results from simulations of an orthogonal frequency division multiplexed (OFDM) radio link. M. Felipe Cátedra of the Universidad de Cantabria (Santander, Spain) presented a personal computer-based tool to predict RF propagation in an urban environment, including up to third-order combinations of reflected and diffracted rays. Tsukasa Iwama of the Communications Research Laboratory (Tokyo, Japan) discussed simulations and field measurements using two-element antennas to enhance propagation for a microcell along a city street. Ami Kanazawa of the Communications Research Laboratory (Tokyo, Japan) presented simulation results and field measurements using an eight-element antenna array to form beams in desired directions.

## 3. Workshop

John Sevic of QUALCOMM, Inc. organized and chair a half-day morning workshop on “Modeling and Simulation of Circuits for Wireless Communication Systems.” Modeling and simulation are essential tools in the rapid and cost-effective design of electronic circuits for wireless applications. Unfortunately, the designers have difficulty in choosing and validating component models. The workshop addressed this issue by offering an introduction to model selection, extraction, validation by measurement, and application. The workshop, attended by 85 people, covered: “Network Analyzer Calibration and Measurement” (Roger Marks, NIST), “Model Selection, Extraction, and Development for Passive Components” (Paul Draxler, QUALCOMM, Inc.), “Simulation of Low-Noise Circuits” (Wayne Struble, M/A-COM, Inc.), “EM Simulation for Passive Elements” (John Dunn, University of Colorado), “Optimization and Statistical Design” (Mike Meehan, Hewlett-Packard EEsof), “Pulsed DC and S-Parameter Extractions for Nonlinear Modeling of High-Power Transistors” (Jaime Plá, Motorola, Inc.), and “Nonlinear Simulation of RF Power Amplifiers for Digital Wireless Systems, (John Sevic, QUALCOMM, Inc.).

## 4. Panel Discussion Session

The rapidly increasing market for wireless services has led to large investments by service providers in spectrum ownership, infrastructure deployment, and service development. In order to probe issues concerning the future evolution of these services and its relationship to technology needs, Sanjay Kasturia organized and moderated an evening Panel Session on “Wireless Evolution and its Impact on Technology Needs.” In a format encouraging audience interaction, the session featured three key technical leaders from the industry: Kari-Pekka Estola, Director of Laboratory of Electronics at Nokia Research Center in Helsinki, Finland; Robert C. Dixon, the former Chief Scientist of Omnipoint Corp. and often considered the “father of spread spectrum technology;” and Paul Anufzkiewicz, Director of RF Engineering with PrimeCo Personal Communications, Westlake, TX.

The discussion brought out the following points:

- Consumer wireless communications systems are forced to accommodate a number of human demands on everything from antenna and base station locations to handset technology. Creative solutions require technical, legal, financial, and sociological awareness. However, operators must be highly innovative to survive in a competitive environment with very high overhead costs in spectrum licenses and infrastructure expenses. In order for the industry to thrive, suppliers must be equally innovative to supply the equipment the service providers need while still driving down costs.
- Personal Communications Services (PCS) are growing rapidly throughout the world. In many places, particularly in the United States, many service providers are planning to operate in the same geographical region. While the systems have been shown functional when operating with a small number of users, many have not been subject to large-scale tests. Furthermore, they have not been shown to be compatible with each other. PCS systems are likely to significantly interfere with each other, particularly since regulations in the United States do not specify guard bands between competing systems. Technical solutions include better filters and amplifiers with better dynamic range.
- Demand for portable wireless data at a high data rate is developing quite slowly.
- The panelists see no major threat to the PCS business in either the future two-way satellite communications systems or the future broadband fixed systems like LMDS.

Near the conclusion of the session Dr. Kasturia posed a question to the audience: did they prefer unregulated spectrum licenses, or would they prefer that government dictate the particular operating standards for their wireless systems? The results came out overwhelmingly in favor of a government mandate concerning the standard. The result was an interesting counterpoint to a keynote address at the 1996 Wireless Communications Conference (via videotape) by Reed Hundt, Chairman of the FCC. Mr. Hundt had emphasized the great strategic advantage the United States had achieved by not adopting such standards. For engineers, the lack of standards, whether government or marketplace imposed, is clearly a great strain.

## 5. Banquet Address

The conference banquet was followed by an address by Michael J. Marcus, Associate Chief of the Office of Engineering and Technology of the FCC. The talk, entitled “Patrolling the Ether: The Search for Captain Midnight,” described Dr. Marcus’s sleuthing adventures in tracking down pirate operators interfering with cable television satellites during the 1980s. For contrast, he also touched on some more constructive approaches to dealing with the FCC.

## 6. Summary

The Wireless Communications Conference is well established as the premier conference in certain interdisciplinary aspects of the field, including the nonlinear behavior of electronics and its impact on digital wireless communications. It is very strong in propagation effects, and it is also becoming an important showcase for broadband wireless communications systems. WCC’97 clearly helped move the industry toward better solutions to its critical technical needs.

## 7. Proceedings

The *1997 Wireless Communications Conference Proceedings* includes 51 papers in 257 pages. The book was distributed at the conference. Copies are available from the IEEE Service Center at +1-908-981-0060 (catalog number 97TH8315).

## 8. Further Information

For further details on the 1997 Wireless Communications Conference, see http://rawcon.org/wcc.

## 9. Future Conferences

The IEEE Microwave Theory and Techniques Society will sponsor the *1998 IEEE Radio and Wireless Conference (RAWCON’98)*, set for August 9–12, 1998 in Colorado Springs, Colorado. Roger Marks is the General Chair and Michael Heutmaker the Technical Program Chair. Information is available on the World Wide Web at http://rawcon.org.

